# Amorphous Fe-Doped Manganese Carbonate for Efficient Activation of Peroxymonosulfate: Mechanism and Performance Toward Orange II Degradation

**DOI:** 10.3390/molecules30112325

**Published:** 2025-05-26

**Authors:** Peng Cheng, Yuqing Li, Yunlong Ma, Cui Qiu, Tengfei Fu, Yajie Wang, Feng Wu

**Affiliations:** 1 Hubei Key Laboratory of Biomass Resource Chemistry and Environmental Biotechnology, School of Resources and Environmental Science, Wuhan University, Wuhan 430079, China; peng.cheng@uca.fr; 2 Institut de Chimie de Clermont Ferrand (ICCF) UMR 6296, Université Clermont Auvergne, Centre National de la Recherche Scientifique (CNRS), Clermont Auvergne INP, BP 80026, F-63171 Clermont-Ferrand, France; 3 School of Eco-Environmental Engineering, Guizhou Minzu University, Guiyang 550025, China; liyuqing@gzmu.edu.cn (Y.L.); mayunlong@gzmu.edu.cn (Y.M.); qiucui@gzmu.edu.cn (C.Q.); 4 Key Laboratory of Coastal Science and Integrated Management, First Institute of Oceanography, Ministry of Natural Resources of the People’s Republic of China, Qingdao 266061, China; futengfei@fio.org.cn

**Keywords:** iron–manganese composite, amorphous material, peroxymonosulfate (PMS), advanced oxidation process (AOP), dye

## Abstract

A novel amorphous Fe-doped manganese carbonate (a-FeMn-1) was synthesized via a facile co-precipitation method and evaluated as an efficient heterogeneous catalyst for the activation of peroxymonosulfate (PMS) in the degradation of Orange II. Among various Fe/Mn molar ratios, the 1:1 composition (a-FeMn-1) showed optimal catalytic activity, achieving 98% removal efficiency within 60 min under near-neutral pH conditions. Characterization results indicated that Fe doping effectively induced an amorphous structure and increased surface area and oxygen defects, promoting PMS activation. The system displayed broad pH applicability and resistance to Cl^−^ and natural organic matter, while degradation was inhibited by HCO_3_^−^ and PO_4_^3−^. EPR and quenching experiments confirmed that surface-bound sulfate radicals (SO_4_^•−^), hydroxyl radicals (^•^OH), and singlet oxygen (^1^O_2_) were the primary reactive species. XPS analysis further revealed the redox cycling of Fe and Mn and the involvement of defect oxygen in the PMS activation process. The catalyst also demonstrated excellent reusability over five cycles without significant loss in efficiency. This work provides insights into the rational design of amorphous bimetallic materials for sulfate radical-based advanced oxidation processes.

## 1. Introduction

Advanced oxidation processes (AOPs) have gained increasing prominence in the degradation of organic pollutants. Sulfate radical (SO_4_^•−^)-based AOPs have attracted significant attention in water treatment [1,2]. SO_4_^•−^ exhibited several advantageous characteristics as a highly reactive oxidant, such as a high redox potential (2.5–3.1 V), a long half-life (30–40 μs), and an exceptional reactivity toward organic compounds containing electron-donating groups [2,3,4]. These properties enable effective and selective degradation of recalcitrant organic contaminants. Both peroxymonosulfate (HSO_5_^−^, PMS) and peroxydisulfate (S_2_O_8_^2−^, PDS) can be activated to generate SO_4_^•−^, along with other reactive oxygen species including hydroxyl radicals, singlet oxygen, and superoxide anions [5]. Due to its asymmetric molecular structure, PMS demonstrates greater activation potential than PDS and can be activated through various approaches, such as ultraviolet irradiation [6], thermal treatment [7], ultrasonic waves [8], base [9], and transition metal activation (Fe, Mn, Cu, Co, etc.) [10,11,12].

Among these activation methods, transition metal-mediated PMS activation offers distinct advantages including operational simplicity, cost-effectiveness, and high efficiency. Previous research has reported that cobalt ions (Co(II)) are the most effective PMS activators [13]. However, Co(II)-based activation is constrained by its leaching issues, as cobalt exhibits significant toxicity even at trace concentrations. Iron ions (Fe(II)) have been widely adopted due to their environmental compatibility and low cost, though their application is limited due to the strict pH requirement and iron sludge formation [14,15]. Other transition metals exhibit relatively lower activation abilities. Therefore, it is essential to develop a transitional metal-based heterogeneous catalyst for the activation of PMS to address the limitation associated with homogeneous catalytic systems.

Among the heterogeneous transition metal catalysts for PMS activation, manganese-based materials have drawn attention due to their multiple valence states (Mn^2+^/Mn^3+^/Mn^4+^), relatively low toxicity, and cost-effectiveness [16,17]. In particular, manganese carbonate (MnCO_3_) has shown promise as a PMS activator owing to its surface redox properties and moderate stability [18]. However, its catalytic performance is often limited by factors such as low surface area, poor dispersion of active sites, and insufficient electron transfer efficiency, especially under neutral pH conditions. These limitations restrict the broader application of MnCO_3_-based materials in advanced water treatment processes.

To overcome these challenges, metal doping strategies have been explored to enhance the catalytic properties of Mn-based materials [19,20,21]. Iron (Fe) doping is particularly attractive due to its synergistic interaction with manganese, which can promote redox cycling, improve electron conductivity, and increase reactive oxygen species (ROS) generation [22]. The incorporation of Fe into MnCO_3_ can potentially enhance the structural disorder and active site density of the material, thereby improving its catalytic efficiency in PMS-based AOPs [23,24].

Recent studies have also highlighted the superior catalytic behavior of amorphous transition metal compounds compared with their crystalline counterparts [25,26]. Amorphous materials typically possess disordered atomic structures, abundant defect sites, and high surface reactivity, all of which contribute to more effective catalyst–oxidant interactions [27]. These features make amorphous metal-based materials promising alternatives to traditional crystalline catalysts [28].

In this study, an iron-doped amorphous manganese carbonate material was synthesized and evaluated as a heterogeneous catalyst for PMS activation in the degradation of Orange II, a representative azo dye, which has been widely used in a large number of industries. Moreover Orange II effluents can be toxic, carcinogenic, mutagenic, or teratogenic [29]. The synergistic effects of iron incorporation and the amorphous structure are expected to enhance the generation of reactive species and improve the degradation efficiency. This work aims to provide a new perspective on the design of high-performance catalysts for sulfate radical-based AOPs through compositional and structural engineering of manganese-based materials.

## 2. Results and Discussion

### 2.1. Characterizations

**Figure 1:**
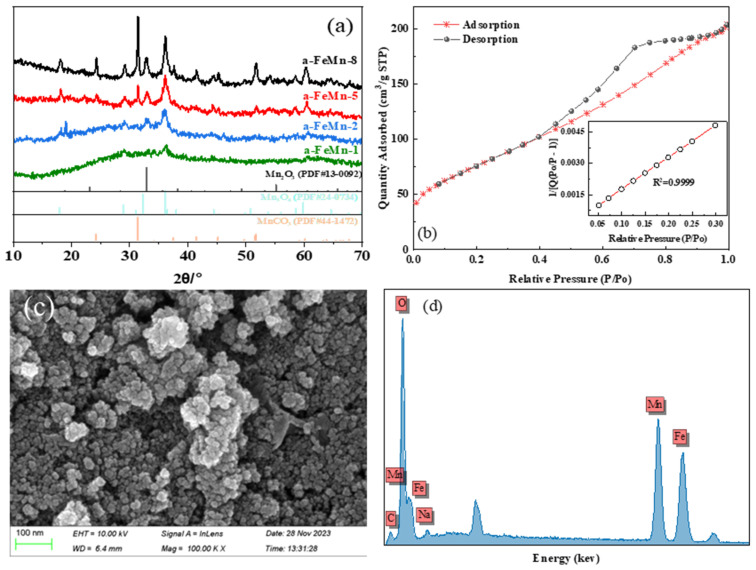
(**a**) XRD pattern of the synthesized Fe-doped manganese carbonate materials with different Fe/Mn ratio; (**b**) N_2_ adsorption–desorption isotherm of a-Fe/Mn-a; (**c**) SEM image of a-Fe/Mn-1; (**d**) energy-dispersive X-ray spectroscopy (EDS) spectrum of a-Fe/Mn-1.

The crystallographic structure of the synthesized Fe-doped manganese carbonate materials was characterized by X-ray diffraction (XRD), as shown in Figure 1a. The sample a-FeMn-8, with the lowest Fe/Mn molar ratio, exhibits sharp and well-defined diffraction peaks corresponding to rhombohedral MnCO_3_ (PDF#44-1472), indicating a high degree of crystallinity. Minor peaks related to Mn_3_O_4_ (PDF#24-0734) and Mn_2_O_3_ (PDF#44-1442) were also observed, indicating partial oxidation valence change during synthesis, which might be due to successful Fe doping. As the Fe content increases (from a-FeMn-8 to a-FeMn-1), the intensity and sharpness of the MnCO_3_ reflections progressively diminish, and the characteristic peaks become increasingly broadened. This trend reflects a gradual loss of long-range crystalline order, which is likely induced by the substitution of Mn^2+^ with Fe^3+^ in the carbonate framework, disrupting the periodic lattice structure.

At the highest Fe/Mn ratio (1:1), the sample a-FeMn-1 displays a broad, featureless diffraction pattern, characteristic of an amorphous phase. The absence of distinct diffraction peaks confirms the successful formation of an Fe-doped amorphous manganese carbonate, which may result from strong lattice distortion and the inhibition of crystal growth due to excess Fe^3^^+^ incorporation. These results demonstrate that increasing Fe doping significantly suppresses the crystallinity of MnCO_3_, ultimately leading to the formation of an amorphous structure at high Fe levels. The structural evolution suggests that Fe incorporation is an effective strategy to tailor the phase and microstructure of Mn-based carbonates. In addition, using the BET method, the obtained amorphous a-FeMn-1 has a large specific surface area, and its specific surface area and pore size are 279.83 m^2^ g^−1^ and 4.51 nm, respectively (Figure 1b).

The SEM images and EDS spectrum of the a-FeMn-1 are presented in Figure 1c,d. SEM image revealed that the a-FeMn-1 possessed a flocculent morphology. Additionally, EDS analysis confirmed the presence of both the Fe and Mn elements, with atomic percentages of 33% and 35%, respectively. The calculated Fe/Mn molar ratio of 1:108 was consistent with the initial stoichiometric ratio.

### 2.2. Catalytic Activity of Fe-Doped Manganese Carbonate with Different Fe/Mn Ratios

To determine the best molar ratio of Fe/Mn of the prepared material for PMS activation, the catalysts with different Fe/Mn molar ratios were used to activate PMS for degrading Orange II at a pH of around 6.0. As shown in Table 1, the a-Fe/Mn-1 exhibited the optimal degradation efficiency of Orange II, reaching up to around after 60 min. The efficiency decreased with a decrease in Fe molar ratio in the composite. The composite with a Fe/Mn molar ratio at 1/8 (defined as a-Fe/Mn-8) showed the lowest catalytic activity, with a degradation of efficiency at 14% after 60 min reaction. In addition, the *k*_obs_ decreased from 0.378 to 0.009 min^−1^ with the molar ratio of Fe/Mn decreasing from 1 to 1/8. This result is likely because the lower Fe/Mn molar ratio negatively affected the redox process of Mn and Fe [30,31]. Given the highest degradation efficiency and *k*_obs_, a-Fe/Mn-1 was selected to be the optimal activator for PMS and degrade Orange II in this work.

### 2.3. The Investigation of Orange II Degradation via a-FeMn-1/PMS System

To investigate the performance of a-FeMn-1-activated PMS in the degradation of Orange II, this study first optimized different parameters, such as the initial pH value, catalyst and PMS dosage. Then, the effects of coexisting ions and natural organic matter (NOM) on the degradation efficiency were studied. Finally, the reusability of the catalyst was tested.

#### 2.3.1. Effect of Different Parameters

**Figure 2:**
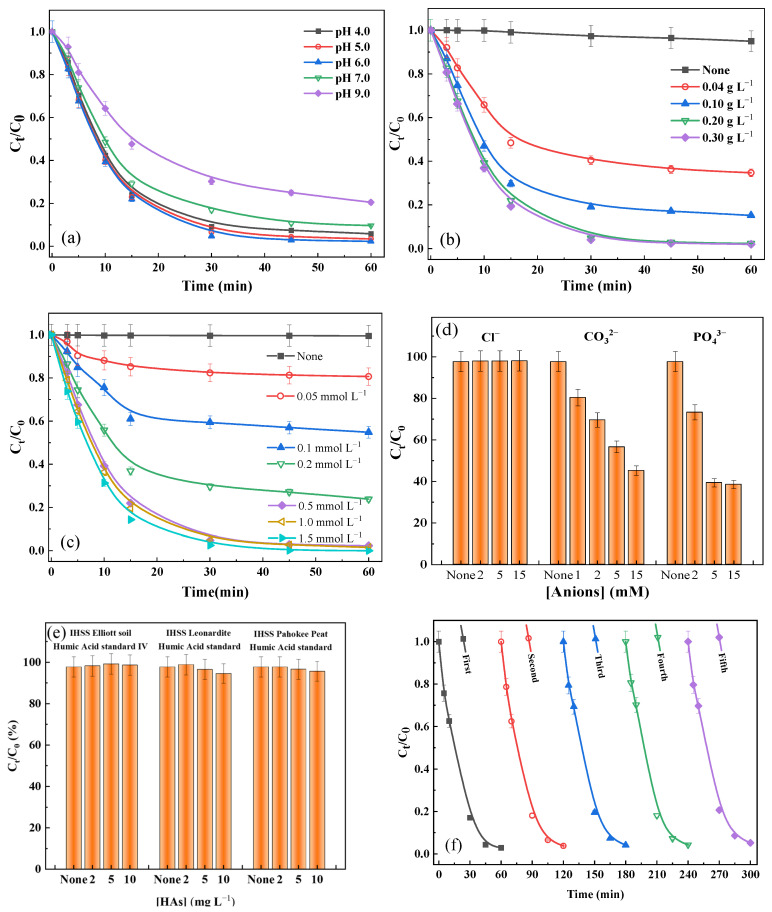
(**a**) The effect of pH on Orange II degradation; (**b**) the effect of a-FeMn-1 dosage on Orange II degradation; (**c**) the effect of PMS concentration on Orange II degradation; (**d**) the effect of co-existing anions on Orange II degradation; (**e**) the effect of NOM on Orange II degradation; (**f**) the Orange II degradation efficiency within 5 reuses of the a-FeMn-1.

The effect of pH on the degradation of Orange II in a-FeMn-1/PMS system is presented in Figure 2a. The system demonstrated an effective removal of Orange II across a broad pH range (4.0–9.0). The degradation of Orange II can reach around 98% at a pH range of 4.0–6.0 and with similar reaction rate constants (*k*_obs_) (0.357 min^−1^, 0.368 min^−1^, 0.378 min^−1^ at pH = 4.0, 5.0 and 6.0, respectively). However, the degradation efficiency of Orange II decreased to 90% and 80% with pH further increasing to 6.0 and 9.0, respectively. The degradation efficiency decreasing as pH increases might be due to the inhibition of activation of PMS by OH^−^ via the impedance of direct contact between a-FeMn-1 and PMS, thereby decreasing active site availability [32]. Additionally, PMS self-decomposition via non-radical pathways at higher pH also induced a lower degradation efficiency of Orange II [33].

The dosage of a-FeMn-1 as an activator significantly influences the removal efficiency of Orange II, as demonstrated in Figure 2b. It is worth noting that nearly no removal of Orange II was found in the absence of a-FeMn-1, indicating that Orange II cannot be directly oxidized by PMS. When the catalyst dosage was increased from 0.04 to 0.20 g L^−1^, the removal efficiency of Orange II improved from 65% to 98% in 60 min, accompanied by an enhancement in *k*_obs_ from 0.1786 to 0.3779 min^−1^. This can be attributed to the increased availability of active sites for PMS activation at higher catalyst loadings. However, a further increase in a-FeMn-1 to 0.30 g L^−1^ cannot enhance the removal efficiency of Orange II as the removal efficiency reaches a plateau. This phenomenon results from the limited PMS concentration in the system [34].

PMS serves as the source of reactive species and plays a pivotal role in Orange II degradation in the a-FeMn-1/PMS system. As illustrated in Figure 2c, the removal efficiency of Orange II exhibited positive correlations with PMS concentrations with a range of 0.05–0.5 mM. Specifically, the removal efficiency increased from 19% to 98% and *k*_obs_ increased from 0.041 to 0.378 min^−1^ as PMS concentration increased from 0.05 mM to 0.5 mM. However, increasing the PMS concentration beyond 0.5 mM led to the stagnation of enhancements in both removal efficiency and *k*_obs_. This arises from the limited active sites on a-FeMn-1for PMS activation and the scavenging effect of excess PMS [34,35].

#### 2.3.2. Effect of Co-Existing Anions and Natural Organic Matter (NOM)

Real wastewater contains different co-existing anions and NOM, which can affect Orange II degradation efficiency. Figure 2d shows the effect of different anions on Orange II degradation. The presence of chloride (Cl^−^) negligibly impacted the removal efficiency of Orange II, while the value of *k*_obs_ exhibited a slight increase from 0.3779 to 0.3852 min^−1^ with rising Cl^−^ concentration. This enhancement can be attributed to the direct reaction between Cl^−^ and HSO_5_^−^, generating Cl_2_ and HOCl, which have been identified as effective oxidants for dye decolorization [36]. In contrast, the presence of carbonate (HCO_3_^−^) significantly inhibited Orange II removal. The removal efficiency decreased from 98% in the absence of carbonate to 45% with 15 mM of carbonate, accompanied by a substantial reduction in *k*_obs_ (from 0.3779 to 0.0085 min^−1^). HCO_3_^−^/CO_3_^2−^ acted as radical scavengers for both SO_4_^•−^ and ^•^OH, with the resultant carbonate radicals (HCO_3_^•^/CO_3_^•−^) exhibiting inferior oxidative capacity when compared with SO_4_^•−^ and ^•^OH, in turn leading to competitive consumption of reactive species [37]. Notably, phosphate (PO_4_^3−^) exerted significant inhibitory effects even at low concentrations (2 mM), reducing the removal efficiency to 73.3% and *k*_obs_ to 0.1971 min^−1^. This might be attributed to the preferential occupation of active sites by PO_4_^3−^ over PMS, decreasing the generation of reactive species. In addition, the formation of less reactive phosphorus-centered radicals (HPO_4_^•−^ and H_2_PO_4_^•^), through reactions with SO_4_^•−^/^•^OH, induces a decrease in removal of Orange II [38].

Moreover, three representative humic acids (HAs) were selected as model natural organic matter (NOM) with which to investigate the effect of NOM on Orange II removal in a-FeMn-1/PMS system. As shown in Figure 2e, the addition of HAs at various concentrations (2, 5, and 10 mg L^−1^) exhibited minimal impact on the removal efficiency of Orange II. However, *k*_obs_ demonstrated bell-shaped profiles across the addition of a range of HA amounts, from 0 to 10 mg L^−1^. The phenolic and quinoid moieties in HAs might catalyze PMS decomposition to generate additional reactive species at lower concentration [39,40]. This system demonstrated an excellent adaptability to natural organic matter.

#### 2.3.3. Reusability of a-FeMn-1

To evaluate the reusability of a-FeMn-1, consecutive removal experiments were conducted by adding identical concentrations of PMS and Orange II to suspension containing 0.2 g L^−1^ a-FeMn-1 at pH 6.0. As shown in Figure 2f, the system maintained excellent performance (98–95%) over five consecutive runs. These results demonstrate the remarkable stability and durability of a-FeMn-1 as a heterogeneous catalyst. The system can effectively achieve continuous Orange II removal through simple procedures, that is, supplementation with fresh PMS and adjustment of pH to 6.0. The minimal efficiency decay (<3% after 5 cycles) confirmed the structural integrity and sustained catalytic activity of a-FeMn-1. In general, the catalyst has good degradation efficiency and stability for Orange II, and its performance is even better than that of some photocatalysts (Table 2).

### 2.4. Identification of the Main Oxidant

**Figure 3:**
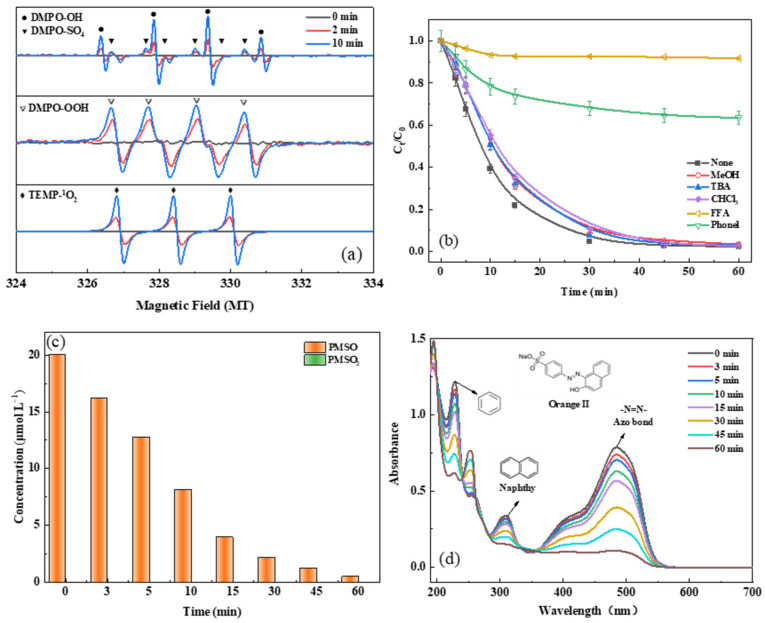
(**a**) EPR spectra at different times in the a-FeMn-1 system; (**b**) the Orange II degradation efficiency in the presence of different scavengers; (**c**) the kinetic of PMSO degradation and PMSO_2_ generation in the a-FeMn-1/PMS system; (**d**) the UV–vis spectra of Orange II at different reaction times in a-FeMn-1/PMS system.

A comprehensive investigation was conducted to identify the reactive species responsible for Orange II oxidation in the a-FeMn-1/PMS system. Electron paramagnetic resonance (EPR) spectroscopy was employed to detect these reactive species, with strong signals corresponding to the DMPO-OH, DMPO-SO_4_, DMPO-OOH and TEMP-^1^O_2_ adducts being observed (Figure 3a), confirming the generation of SO_4_^•−^, ^•^OH, O_2_^•−^, and ^1^O_2_ in the experimental system.

To quantify the contribution of different reactive species, quenching experiments were conducted to identify the predominant oxidants responsible for Orange II degradation (Figure 3b). Isopropanol (IPA) can effectively quench SO_4_^•−^ and ^•^OH with second-order rate constants of $k_{IPA,{SO}_{4}^{•-}}$ = 8.2 × 10^7^ M^−1^∙s^−1^ and $k_{IPA{,}^{•}OH}$ = 1.9 × 10^9^ M^−1^∙s^−1^, whereas *tert*-butanol (TBA) can only quench ^•^OH ($k_{TBA{,}^{•}OH}$ = 6.0 × 10^8^ M^−1^∙s^−1^; $k_{TBA,{SO}_{4}^{•-}}$ = 8.3 × 10^5^ M^−1^∙s^−1^) [40,44,45,46]. In the system of a-FeMn-1/PMS, neither IPA nor TBA had an effect on the degradation of Orange II. This phenomenon is obviously contrary to the results of EPR, in which the typical DMPO-OH and DMPO-SO_4_ signals are observed under the same conditions. This is possibly due to the high dielectric constants of IPA (18.0) and TBA (17.8), which make them difficult to assemble on the surface of the solid [41]. Therefore, both can only quench radicals in a solution but not on a solid surface. To verify this hypothesis, phenol was selected as the scavenger for both SO_4_^•−^ and ^•^OH in the solution and at the surface of the solid, because it has a low dielectric constant of 8.0 and a high reactivity toward SO_4_^•−^ and ^•^OH ($k_{phenol,{SO}_{4}^{•-}}$ = 6.6 × 10^9^ M^−1^∙s^−1^ and $k_{phenol{,}^{•}OH}$ = 8.8 × 10^9^ M^−1^∙s^−1^) [42]. As shown in Figure 3b, the addition of 5.0 mM phenol can almost completely inhibit the degradation of Orange II, with the efficiency decreasing from 98% to 36% within 60 min. Therefore, SO_4_^•−^ and ^•^OH mainly presented at the surface of the a-FeMn-1 and the oxidation of Orange II correspondingly occurred at the surface of the catalyst.

Chloroform (CHCl_3_) is an effective scavenger for O_2_^•−^ due to its high reactivity (k = 1.6 × 10^10^ M^−1^ s^−1^) [42]. A negligible effect was observed with 5.0 mM of CHCl_3_, suggesting minimal contribution from O_2_^•−^. Meanwhile, furfuryl alcohol (FFA), primarily serving as a ^1^O_2_ scavenger, exhibits additional quenching capabilities toward both SO_4_^•−^ and ^•^OH. As demonstrated in Figure 3b, the presence of 5.0 mM FFA resulted in only a 9.2% degradation efficiency, which is due to the contribution of the SO_4_^•−^/^•^OH and ^1^O_2_. This observation might initially suggest that ^1^O_2_ plays a dominant role in Orange II degradation, which may be due to the presence of a large number of lattice defects in the amorphous complex, such as oxygen vacancies, which can activate PMS to produce singlet oxygen rather than sulfate radicals [43]. Additionally, no PMSO_2_ production was detected upon addition of 20 μM PMSO in the system, confirming the absence of high-valent metal species, as shown in Figure 3c [47]. These results collectively demonstrate that Orange II degradation predominantly occurred via surface-mediated SO_4_^•−^/^•^OH and ^1^O_2_.

The UV–vis spectra of Orange II were monitored during the reaction (Figure 3d). The characteristic absorbance at 484 nm (azo bond), along with peaks at 228 nm (naphthalene ring) and 309 nm (benzene ring), showed progressive attenuation, indicating direct attack on the azo linkage by reactive species [48]. Concurrent decrease of aromatic ring-associated peaks suggested structural fragmentation, while the emerging peak at 251 nm implied the formation of intermediated products.

### 2.5. XPS Analysis

**Figure 4:**
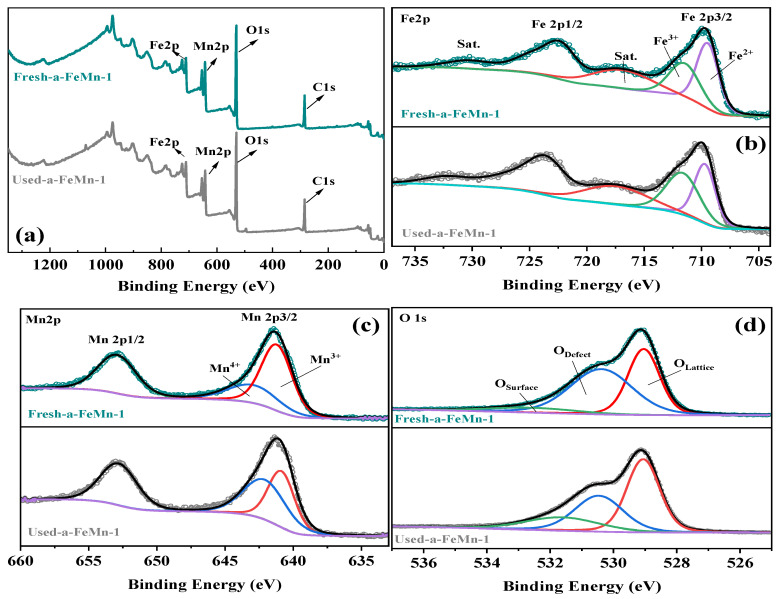
XPS spectra of a-FeMn-1 before and after the reaction: (**a**) Full survey; (**b**) Fe 2p spectra; (**c**) Mn 2p spectra; (**d**) O 1s spectra.

To investigate the surface chemical state changes of active components during the degradation of Orange II, X-ray photoelectron spectroscopy (XPS) was employed to systematically analyze the oxidation states of Fe, Mn and O on the a-FeMn-1 surface. As shown in Figure 4a, the full survey spectra of a-FeMn-1 before and after the reaction exhibited no significant changes, suggesting good structural stability of the catalyst under operating conditions.

High-resolution Fe 2p spectra (Figure 4b) of fresh a-FeMn-1 displayed two distinct peaks at 710.38 eV and 723.68 eV, corresponding to Fe 2p3/2 and Fe 2p1/2, respectively [49]. Deconvolution of the Fe 2p3/2 peak revealed two components located at 710.44 eV (Fe^2^^+^) and 712.65 eV (Fe^3+^), with relative contents of 57.2% and 42.8%, respectively [49,50]. This indicates partial reduction of Fe^3^^+^ during synthesis, possibly due to the presence of Mn^2+^ and electron transfer between the transition metal ions. After the reaction, the Fe 2p3/2 peak slightly shifted toward higher binding energy and the proportion of Fe^2+^ decreased to 47.4%, while Fe^3^^+^ increased to 52.6%, suggesting oxidation of Fe during the catalytic process.

The Mn 2p spectra of fresh and used samples are shown in Figure 4c. The fresh sample exhibited two main peaks at 641.8 eV and 653.5 eV, corresponding to Mn 2p3/2 and Mn 2p1/2, respectively. Notably, no satellite peak was observed near 647 eV, indicating the absence of Mn^2+^ and implying that Mn^2+^ was fully oxidized during synthesis, possibly due to the presence of dissolved oxygen and Fe^3^^+^ in the solution [51]. The Mn 2p3/2 peak could be deconvoluted into two components located at 641.5 eV (Mn^3^^+^) and 643.3 eV (Mn^4+^), confirming the coexistence of mixed-valence manganese species. After the reaction, the relative content of Mn^4+^ increased, indicating that Mn also participated in redox transformations during the activation of PMS.

As shown in Figure 4d, the O 1s spectra could be fitted into three components located at 529.0 eV, 530.4 eV and 532.6 eV, which are attributed to lattice oxygen (O_lattice), oxygen vacancies or defect oxygen (O_defect), and surface-adsorbed oxygen species (O_surface), respectively [52]. The fresh sample exhibited a high proportion of defect oxygen (52.8%), consistent with the amorphous nature of the material. After the reaction, this proportion decreased to 39.2%, suggesting that defect oxygen was actively involved in PMS activation, possibly contributing to the generation of singlet oxygen (^1^O_2_), in agreement with ESR and quenching experiment results [53,54]. 

## 3. Materials and Methods

### 3.1. Chemicals

Potassium peroxymonosulfate (KHSO_5_·0.5KHSO_4_·0.5K_2_SO_4_), 5,5-Dimethyl-1-pyrroline N-oxide (DMPO), and 2,2,6,6-tetra-methyl-piperidine (TEMP) were purchased from Sigma Aldrich. Orange II (C_16_H_11_N_2_NaO_4_S, >85.0%), manganese(II) nitrate tetrahydrate (Mn(NO_3_)_2_·4H_2_O, 98%), and iron(III) nitrate nonahydrate (Fe(NO_3_)_3_·9H_2_O, 99%) were purchased from Shanghai Macklin Biochemical Technology Co., Ltd. (Shanghai, China). Humic acids (HAs) were purchase from the International Humic Substances Society (IHSS). The dissolution of HAs was performed by first dissolving in 0.01 M NaOH, with subsequent adjustment to pH 7.0 using 0.1 M HClO_4_ under continuous ultrasound assistance. All other reagents in this work were of analytical grade or higher and used without further purification. All solutions were prepared using Milli-Q water (Guangzhou Baiwei Instrument Technology Co., Ltd., Shanghai, China) (>18.25 MΩ cm, DOC < 0.1 mg L^−1^).

### 3.2. Synthesis of Fe-Doped Amorphous Manganese Carbonate

Fe-doped amorphous manganese carbonate was synthesized via a co-precipitation method. Initially, Fe(NO_3_)_3_·9H_2_O (1.0 mmol) and Mn(NO_3_)_2_·4H_2_O (1.0 mmol) were dissolved in 100 mL of Milli-Q water under constant stirring for 30 min to form solution A. Separately, 0.035 mol of NaOH and 0.015 mol of Na_2_CO_3_ were dissolved in another 100 mL of Milli-Q water to obtain solution B. Solution B was added dropwise into solution A under constant stirring, while maintaining the pH at 10.5 ± 0.2 throughout the addition process. After complete mixing, the resulting suspension was subjected to ultrasonication for 30 min, followed by magnetic stirring in a 65 °C water bath for 4 h to ensure complete precipitation and uniform particle formation. The resulting solid was collected by centrifugation and filtration, then thoroughly washed with Milli-Q water 6–8 times to remove residual ions. The washed product was dried in a vacuum oven at 60 °C for 24 h and stored in a desiccator for further characterization.

To investigate the effect of iron doping, a series of samples was synthesized by varying the Fe/Mn molar ratios to 1:1, 1:2, 1:5 and 1:8, while keeping the total amount of metal ions constant at 2.0 mmol. All other synthesis conditions remained unchanged. The resulting materials were labeled as a-FeMn-1, a-FeMn-2, a-FeMn-5, and a-FeMn-8, corresponding to Fe/Mn molar ratios of 1:1, 1:2, 1:5, and 1:8, respectively.

### 3.3. Catalyst Characterization

An X-ray diffractometer (XRD, D-8 Bruker, Karlsruhe, Germany) was employed to investigate the crystal structure of the catalyst. The chemical function group on the catalyst’s surface was analyzed by Fourier transfer infrared spectrometer (FTIR, Thermo Fisher Scientific 4700, Waltham, MA, USA). X-ray photoelectron spectroscopy (XPS, Thermo Fisher Scientific K-Alpha, Waltham, MA, USA) was used to analyze the chemical states of elements on the solid’s surface. The morphology of the catalyst was observed using a scanning electron microscope (SEM, Zeiss Sigma 300, Oberkochen, Germany). The composition and content of samples were analyzed using energy dispersive spectrometry (EDS, XFlash6I30, Karlsruhe, Germany). The specific surface area of the samples was investigated using Brunauer–Emmett–Teller analysis (BET, ASAP 2460, Norcross, GA, USA).

### 3.4. Experiment Procedure

The experiments were conducted at room temperature (25 ± 2 °C) in 250 mL brown glass vessels. A mixed reaction solution with a specific concentration of Orange II and PMS was prepared. The initial pH of the reaction solution was then adjusted using HClO_4_ or NaOH solutions. Reactions were initiated by adding a-FeMnC powders. Samples were withdrawn using a syringe at specified intervals, then filtered through a 0.22 μm filter. The residual concentration of Orange II was measured at 484 nm using a UV759 UV–Vis spectrophotometer. To obtain the optimal experiment conditions, the effect of pH (4.0, 5.0, 6.0, 7.0 and 9.0), a-FeMn-1 dosage (0.04–0.30 g L^−1^), PMS (0.05–0.15 mM), different co-existing anions (Cl^−^, HCO_3_^−^, PO_4_^3−^) at different concentrations (2, 5, 15 mM) and different humic acids (Elliott Soil humic acid standard, Pahokee Peat humic acid standard, and Leonardite humic acid standard) at different concentrations (2, 5, 10 mg L^−1^) were investigated. To determine the contribution of different reactive species for Orange II degradation, quenching experiments were conducted. Isopropanol (IPA), *tert*-Butanol (TBA), chloroform (CHCl_3_), phenol and furfuryl alcohol (FFA) were added to the suspension at a concentration of 5 mM. To evaluate the recyclability of a-FeMn-1, the catalysts were reused for 5 cycles in sequential experiments without any regeneration treatment.

### 3.5. Analysis Methods

The concentration of Orange II was determined at a wavelength of 484 nm by using UV–Vis spectrometry. The variation of PMS concentration was measured using iodine dosimetry [55]. The leaching of ferrous ions and total iron in the system was determined using the Ferrozine method [56]. The generation of radicals and ^1^O_2_ was detected using electron paramagnetic resonance spectrometer (EPR, JES-FA300, Japan) with 5,5-dimethyl-1-pyrroline-N-oxide (DMPO) as a spin trapping agent for SO_4_^•−^, ^•^OH, and O_2_^−^. Additionally, 2,2,6,6-tetramethyl-4-piperidine (TEMP) was used as an ^1^O_2_ trapping agent in the a-FeMn-1/PMS system. The generation of high-valence metal in this system was determined using methyl phenyl sulfoxide (PMSO) as a probe, while the consumption of PMSO and the generation of methyl phenyl sulfone (PMSO_2_) were analyzed by HPLC [57].

To compare the reaction rate constants under different experimental conditions, a pseudo-first-order equation was used to fit the experimental data. The pseudo-first order equation can be described as follows:

(1) $$\ln C_{t}/C_{0}=-k_{obs}\;t$$

where C_0_ and C_t_ are the Orange II concentrations at the initial time and any reaction time t, respectively. *k*_obs_ (min^−1^), namely apparent reaction rate constants, was calculated from the slope of the plots of ln (C_t_/C_0_). Considering the complexity of the mechanisms and the fast PMS consumption in the system, the equation was applied to the data obtained within the initial 15 min.

## 4. Conclusions

In this study, an amorphous Fe-doped manganese carbonate (a-FeMn-1) catalyst was successfully synthesized and applied for the efficient degradation of Orange II via peroxymonosulfate activation. The co-presence of Fe and Mn in an amorphous matrix resulted in enhanced surface area, high oxygen defect density, and synergistic redox activity, facilitating the generation of SO_4_^•−^, ^•^OH and ^1^O_2_. The system performed well under neutral pH conditions and maintained catalytic activity in the presence of chloride ions and natural organic matter, while bicarbonate and phosphate significantly inhibited the reaction. Mechanistic studies confirmed that surface-mediated radical oxidation was the dominant degradation pathway. Furthermore, a-FeMn-1 exhibited excellent stability and reusability over five consecutive cycles. This work highlights the potential of amorphous bimetallic carbonate catalysts in environmental remediation and offers a promising strategy for the development of sustainable advanced oxidation processes.

## Figures and Tables

**Table 1:** The degradation efficiency of Orange II with different prepared material.

Materials	pH	[PMS] (mM)	[Catalyst] (g L^−1^)	[Orange II] (μM)	Removal Rate	*k*_obs_ (min^−1^)
a-FeMn-1	6.0	0.5	0.2	50	97.7%	0.378 ± 0.001
a-FeMn-2	6.0	0.5	0.2	50	87.5%	0.079 ± 0.002
a-FeMn-5	6.0	0.5	0.2	50	80.0	0.054 ± 0.003
a-FeMn-8	6.0	0.5	0.2	50	14.0	0.009 ± 0.001

**Table 2:** Comparison of Orange II degradation performance using different catalysts.

Material	pH	Time min	[Catalyst] g/L	[PMS] mM	[Orange II] μM	Removal Rate	Ref
a-FeMn-1	6.0	30	0.2	0.5	50.0	95.0%	This study
WPS-Fe-350 ☉	3.0	80	1.0	-	85.7	83.0%	[41]
Zn-Al-LDO ☉	-	100	0.1	-	210.0	74.3%	[42]
Fe/Mn/Mg2-LDH	7.0	30	1.6	1.0	50.0	98.0%	[4]
om-MnFe_2_O_4_	-	30	0.2	2.0	143	97.3	[43]

☉ Represents the photocatalyst.

## Data Availability

All data discussed in the paper are provided either within the article. Raw data files will be provided upon request.
